# Clone journals: a threat to medical research

**DOI:** 10.1590/1516-3180.2018.0370160919

**Published:** 2020-03-06

**Authors:** Zeeshan Asim, Shahryar Sorooshian

**Affiliations:** I PhD. Doctoral Student, Faculty of Industrial Management, Universiti Malaysia Pahang, Kuantan, Malaysia.; II PhD. Senior Lecturer, Department of Business and Administration, School of Business Economic and Law, University of Gothenburg Gothenburg, Sweden.

Dear Editor,

The scholarly world is currently facing various anomalous threats that include paid publishers and fake journals. There has been enough debate about the challenges of predatory publishers, which encourage authors to publish their work in unreliable peer-reviewed journals, for a price.

A new discussion has spread across the scholarly world regarding fake journals. This is a recent phenomenon of even more malevolent fraud that has broken into the realm of the academic world. This phenomenon is also commonly known as “hijacked journals” or “clone journals.”

Clone journal web pages are a counterfeit mirror of an authentic journal that exploit the title and ISSN of legitimate journals.^1^ In contrast to predatory journals, clone journals are more likely to accept papers from authors, since they have developed as the mirror image of reputable journals, including their domain name. Usually, they receive massive attention through claiming that they have earned high impact factors from reputable indexing agencies such as Web of Science and Scopus.^1^

Some of these counterfeit journals actively chase authors by trawling through the latest conference proceedings to acquire the e-mail addresses of participants. These people are then approached through a modified e-mail message that announces a fake call for papers in a current issue of the journal. Careless authors may be duped by these solicitations into paying an open-access publication fee, trusting that their work is about to be published in a reputable journal.

Once these authors have paid the publication fee, they may lose ownership over their submission because they will have signed it over to the clone publisher. Consequently, they will be unable to get a refund or withdraw the article from publication. Since the article has now been “officially” published, it no longer meets the legitimate criteria of most reputable journals, which have ethical guidelines regarding the submission process, which include a declaration that the article has not previously been published. In this regard, clone journals present a serious threat to the integrity of scientific publishing and place a big question mark on the peer review process.

The main apprehension regarding this new trend is that unreviewed manuscripts that are published on these clone websites may become sources of medical practice and health policy and might be incorporated into systematic reviews on the clinical literature.^2^ Another major concern that these clone journals may impose on clinical sciences is that their unreviewed outcomes could become sources for novel hypotheses. These can be considered to be a severe threat to the reliability and validity of future medical research.^2^

The prominent journals relating to medical science that have fallen victim to this phenomenon over time have included *“Revista Brasileira de Medicina do Esporte (RBME)”, “Emergencies”, “Journal of the American Medical Association”, “Vitae Revista”, “Terapevticheskii Arkhiv”* and *“Kardiologiya”*.^3^ Dadkhah and Borchardt inspected 2,442 papers in just five clones of journals in which researchers and scientist thought that they had published their articles during the first quarter of 2015.^4^ According to their report, there are various unknown clone journals that continuously transform their web domain addresses. The cybercriminals behind this phenomenon seem to develop new clone journals every day, thus targeting increasing numbers of authors who may be unaware of this threat.^4^

Many researchers have highlighted a range of threats to the reliability of the scientific process of deceitful publishers, but none of them have addressed how these cybercriminals process their clone mechanism. Although publishers pay their registration charges for web domain services on a regular basis, any failure to do so could allow a waiting cyber intruder to swoop in and steal a domain for their own purposes and, at the same time, divert the entire web traffic towards the clone journal website. In the cyber world, the phenomenon of stealing web domains is known as “web swooping”.^5^

In conclusion, issuing warnings to the world of medical sciences regarding this new threat and conducting rigorous scientific reviews of citations to and from medical research articles could form the most realistic measures for combating this phenomenon. The council of science editors has suggested some cautionary red flags with the aim of educating the worldwide scholarly publishing community before sending manuscript submissions.^6^ These include the need for authors to be aware of the following, regarding clone journals: (1) False claims to be members of the Committee on Publication Ethics (COPE) and the Open Access Scholarly Publishing Association (OASPA); (2) False declarations of indexation in databases such as SCOPUS and Web of Science (WOS); (3) Manuscript publication charges that are not visible; (4) Non-transparency of the peer-review process, with unrealistically short peer review-to-publication turnaround times (e.g. one week); (5) Non-existent publisher contact details: fake publishers do not have authentic postal addresses or any active telephone number; and (6) Counterfeit publishers have small numbers of articles per year but have enormous editorial boards, or vice versa.

Nonetheless, these precautionary steps require long-term measures that would enable use of stricter and more advanced techniques with the aim of eliminating these threats, along with effective copyright measures that can protect the reliability and validity of published medical science articles.
