# ERRATUM

**DOI:** 10.1590/1516-3180.2018.ZA.037018022020erratum

**Published:** 2020-04-22

At the request of the authors, we report that in the paper published in the Sao Paulo Medical Journal, volume 137, issue number 6, DOI: 10.1590/1516-3180.2018.0370160919, page 550:

Where it read:

“^I^PhD. Doctoral Student, Faculty of Industrial Management, Universiti Malaysia Pahang, Kuantan, Malaysia”

It should read:

“PhD. Assistant Professor, Electrical Engineering Department, Institute of Business Management (IoBM), Karachi, Pakistan”

